# Item response theory and validity of the NEO-FFI in adolescents

**DOI:** 10.1016/j.paid.2012.06.002

**Published:** 2012-10

**Authors:** Ruth Spence, Matthew Owens, Ian Goodyer

**Affiliations:** Department of Psychiatry, Cambridge University, UK

**Keywords:** Personality, Adolescents, Item response theory, External validity

## Abstract

The present study applied item response theory (IRT) to the NEO five factor inventory (NEO-FFI) completed by a community based sample of adolescents. The results revealed that many of these personality items may not be discriminating well, with some traits demonstrating greater reliability than others. Furthermore, the threshold values highlighted that the majority of the items had skewed responses, suggesting a limited utility of some response categories. Generally, removing poorly discriminating items does not harm external validity, suggesting IRT reduces measurement error and increases reliability without compromising validity.

## Highlights

► It is important to maximise the precision of personality measurement in adolescents. ► We apply item response theory (IRT) to the NEO-FFI in an adolescent sample. ► IRT was used to assess item validity and highlight poorly performing indicators. ► Removing poor items reduced measurement error without compromising validity. ► IRT analysis can be used to develop personality measures ensuring item validity.

## 1 Introduction

The five factor model is one of the most extensively applied models of personality currently in use. The personality traits of Neuroticism, Extraversion, Openness, Agreeableness and Conscientiousness have been repeatedly found across ages, cultures and within the same individual over time (e.g. De Fruyt, De Bolle, McCrae, Terracciano, & Costa, 2009; Hřebíčková et al., 2002; McCrae, Costa, & Martin, 2005). This has led to them being empirically related to a cornucopia of concepts as well as used in mediation and moderation models of current behaviours, helping to define relationships and explain outcomes. In adolescence, personality may even be a key mediator of individual differences in the course and treatment responses of youth with mental disorders that emerge at this period in development (Costello, Copeland, & Angold, 2011).

However, on closer inspection, problems remain with personality measurement in adolescents. In comparison to adult research, studies with adolescents have found more cross loadings, and items that do not load sufficiently on any factor. Additionally, the studies demonstrate that items from the Neuroticism and Conscientiousness scales perform better, whereas Extraversion, Agreeableness and Openness items have less reliability (e.g. Parker & Stumpf, 1998; Sneed, Gullone, & Moore, 2002). The problems with factor replicability may be due to developmental changes that take place during this time; personality traits are still in flux throughout adolescence (McCrae et al., 2002) and the structure and coherence of the five factors vary at different ages (Soto, John, Gosling, & Potter, 2008). Therefore it is important to determine if the precision of personality measurement can be maximised for use in behavioural and clinical studies in this age range.

Item response theory (IRT) can be used to improve the measurement of adolescent personality. The application of IRT allows scale psychometric properties to be revealed with greater precision than other multivariate methodologies; analysing item level information can provide insights into measurement reliability and enables a thorough evaluation of the internal construct validity.

IRT provides information by checking the validity of the items and delineating poor performing indicators. It does this by estimating each individual item’s discrimination on the latent trait (the *a* parameter) and difficulty within a population (the *b* parameter) (Embretson & Reise, 2000). An item’s discrimination reflects how the probability of endorsing an item changes as the level of the underlying trait increases. Thus, highly discriminating items more strongly represent the latent trait. The item’s difficulty corresponds to the likelihood of an individual endorsing it given their level of the latent trait. An item is considered easy if most people endorse it and the difficulty rises as the likelihood of endorsing it decreases. Therefore some items may be easy to endorse even at relatively low levels of the latent trait. IRT also provides estimates of each scale and item’s total information function through total and item information curves (TICs and IICs). These depict the amount of information provided across levels of the latent trait. This means IRT can be used to reveal how informative a measure is at all levels of the latent trait (Baker, 2001).

Although IRT can be used to assess the internal validity of a measure, correlates are needed to examine the impact on external validity. IRT analysis has been conducted with adult samples and shows that when the best performing items are chosen, shortened versions of personality inventories often have similar predictive capabilities (Thalmayer, Saucier, & Eigenhuis, 2011). Indeed, Reise and Henson (2000) found that after IRT the NEO-PI-R could be greatly reduced and for many scales only the best four items were needed to produce comparable facet results. Such psychometric research has however not been carried out with younger populations.

As well as delineating internal construct validity this study uses several measures to examine external criterion validity including educational performance, current friendships and general well-being. These measures cover the domains of adolescent competence which are important for the successful negotiation of developmental tasks (Masten et al., 1995). Each personality trait is hypothesised to correlate to varying degrees with the different facets of adolescent competence and therefore go some way towards highlighting a personality pattern associated with individual differences in competent adolescent functioning.

It is hypothesised that Extraversion and Conscientiousness will be positively and Neuroticism negatively associated with well-being (Siegler & Brummett, 2000). Likewise, elevated levels of Conscientiousness and Openness will be associated with school performance (Chamorro-Premuzic & Furnham, 2003; De Fruyt, van Leeuwen, de Bolle, & de Clercq, 2008). Finally we will examine whether Extraversion and Agreeableness are associated with the quality of current friendship (Scholte, van Aken, & van Lieshout, 1997; Selfhout et al., 2010).

This study applies IRT methodology to the NEO-FFI in order to investigate how it can be utilised to improve the validity of personality measurement in a late adolescent population. Furthermore, an examination of external validity will explore which personality traits are associated with adolescent competence as indexed by measures of current well-being, friendships and school examination performance.

## 2 Methods

### 2.1 Participants

Participants were 470 English adolescents (295 females, 175 males) who completed the NEO-FFI; mean age 18.7 years (age range: 17.7–20.2 years, SD = 0.55). The participants are part of the ongoing ROOTS study; a longitudinal study of 1204 participants aged 14 years at first recruitment and reassessed at 15.5 and 17.5 years (Goodyer, Croudace, Dunn, Herbert, & Jones, 2010). At 17.5 years data were gathered about academic achievement; additionally participants completed a friendship satisfaction questionnaire (Goodyer, Wright, & Altham, 1989) and the Warwick–Edinburgh Mental Well-being Scale (WEMWBS; Tennant, Fishwick, Platt, Joseph, & Stewart-Brown, 2006). The self-report version of the NEO-FFI was sent via post an average of 14.9 months after the other measures were completed (range: 5.2–28.6 months, SD = 6.1). Questionnaires were returned by 470 (43.8%) of the remaining sample and complete for 438 (36.4% of the cohort and 93.2% of the questionnaire responders) of this sub-sample.

### 2.2 Measures

The NEO-FFI was developed from the NEO-PI-R (Costa & McCrae, 1992). The NEO-PI-R contains 240 items measuring five domains (Neuroticism, Extraversion, Openness, Agreeableness and Conscientiousness) represented by specific facets (e.g. Neuroticism is measured by items covering hostility, depression, self-consciousness, impulsiveness, vulnerability to stress and anxiety). The NEO-FFI contains 60 items which are summed to measure personality at the domain level only. Each item consists of a statement rated on a Likert scale ranging from strongly disagree to strongly agree. Scale alpha reliabilities for this sample were .88 (Neuroticism), .81 (Extraversion), .74 (Openness), .77 (Agreeableness) and .87 (Conscientiousness).

The WEMWBS (Tennant et al., 2006) is a self report measure of well-being covering two distinct perspectives. The hedonic perspective focuses on the subjective experience of happiness and life satisfaction, and the eudaimonic perspective, focusing on psychological functioning and self realisation. The measure consists of 14 positively worded items asking about thoughts and feelings over the previous 2 week period, each scored from 1 ‘none of the time’ to 5 ‘all of the time’. Scale alpha reliability was 0.89.

The friendship satisfaction questions (Goodyer et al., 1989) were taken from a semi-structured interview schedule enquiring about components of peer relationships over the last 12 months. There are eight questions, incorporating three features of the relationships; availability, adequacy and intimacy, to provide a global rating of friendship. Items asking about frequency of occurrences (e.g. do your friends tease you?) are rated from 0 ‘never’ to 5 ‘almost every day’, whereas questions about satisfaction of friendships (e.g. can you confide in your friends?) are rated from 0 ‘not at all’ to 3 ‘most of the time’. Scale alpha reliability was 0.71.

Data were collected regarding the general certificate of secondary education (GCSE). This is an academic qualification awarded in a specific subject, such as English or Maths, usually taken by students aged between 14 and 16 years. Generally each student is entered for examination on between 8 and 10 subjects, although this is subject to variation. The highest pass grade awarded is an A^∗^ continuing down to grade G. The number of GCSE entries, plus the number of GCSE qualifications each participant achieved at grades A^∗^–C and D–G were used as reflecting indices of school performance.

### 2.3 Analysis

The IRT analysis used a graded response model (Samejima, 1969), which is appropriate for ordered categorical responses such as the Likert scales used by the NEO-FFI. This model also allows the individual items to have a different number of response categories. IRT assumes local independence of the items and unidimensionality of each of the factors. Unidimensionality was assessed using confirmatory factor analysis (CFA) where the items were specified to load on one factor. Currently, there is no standard procedure for establishing adequate unidimensionality, generally evidence of a dominant factor explaining a large proportion of the variance and goodness of fit indices (GFIs) are assessed (Embretson & Reise, 2000).

Analysis was conducted in the M*plus* Programme (Version 6, Muthén & Muthén, 1998–2010). IRT was performed using an MLR estimator and a logit link, which sets the scale to use log metric. Baker (2001) produced guidelines for judging item discrimination levels, moderate discrimination is achieved if the *a*-parameter is between .65 and 1.34 and high discrimination if the *a*-parameter is 1.35–1.69. A value halfway between these two ranges was chosen to signify items having moderate to high discrimination, thus a cut off of *a* > 1.17 was used.

The factor scores for each personality scale were correlated using Pearson correlations with the well-being and friendship measures and regressed onto the academic achievement variables before and after IRT. The non-IRT and IRT correlations and regressions were compared using Steiger’s *z*-test. This assesses whether relationships found from the same population are significantly different.

## 3 Results

The trait means were compared to the college-age (17–21 years) norms given in the NEO-FFI manual (Costa & McCrae, 1992). The sample was lower on Neuroticism (*t*(842) = 6.15, *p* < .001) and higher on Agreeableness (*t*(844) = 5.36, *p* < .001) but not significantly different on Extraversion (*t*(847) = 0.34, *n.s.*), Openness (*t*(847) = 1.16, *n.s.*) or Conscientiousness (*t*(846) = 0.64, *n.s.*).

### 3.1 IRT analysis

The unidimensionality assessment revealed the GFIs for one factor models were not good. Additionally, each scale had moderately correlated residuals between the items; the NEO-FFI scales contain items that represent the different NEO-PI-R facets to varying degrees, likely causing this inter-item covariation. Therefore modification indices were used to include item correlations improving model fit (see Table 1).

Bi-factor models were used to model the multidimensionality within the data. Bi-factor models allow the scale items to load on the dominant latent trait underlying all the items, additionally items can load on one or more narrower ‘group’ factors, providing a way to fit multi-dimensional IRT models (Reise, Morizot, & Hays, 2007).

**Fig. 1:**
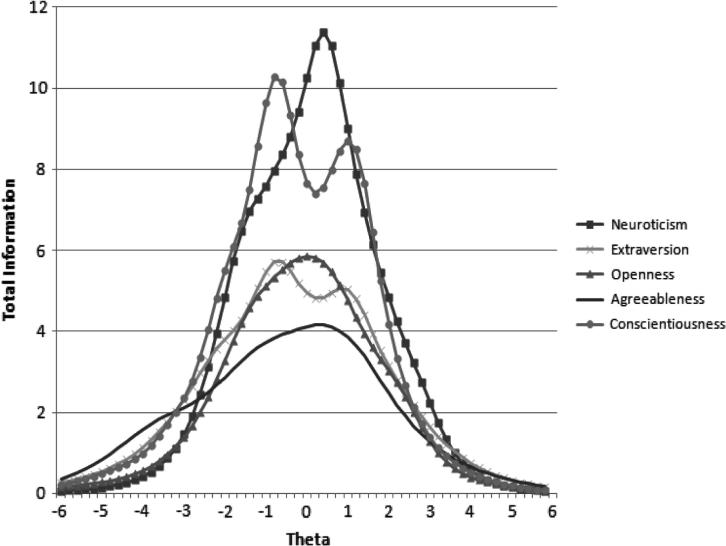
Bifactor model total information curves for each of the 12-item personality scales.

IRT revealed each scale had items that did not achieve moderate to high discrimination on the general factor (see Table 2). The scales achieved their greatest precision within ± one standard deviation from the mean level of the trait. This is to be expected given the instrument was designed to measure normative trait levels. Specifically, the TICs peaked around 0.4 for Neuroticism, peaked once around −0.8 and again around 0.8 for Extraversion, around 0.0 for Openness, around 0.4 for Agreeableness and peaked twice for Conscientiousness, once around −0.8 and once around 1.0 (see Fig. 1).

The threshold data revealed that to endorse the response category of “*strongly disagree*” an individual had to lie beyond three standard deviations from the mean for 51 (85%) of the items, with a further 7 (11.7%) items having no-one endorse this option. Furthermore, individuals had to score above three standard deviations from the mean for 26 (43.3%) items to reply “*strongly agree*”.

**Fig. 2:**
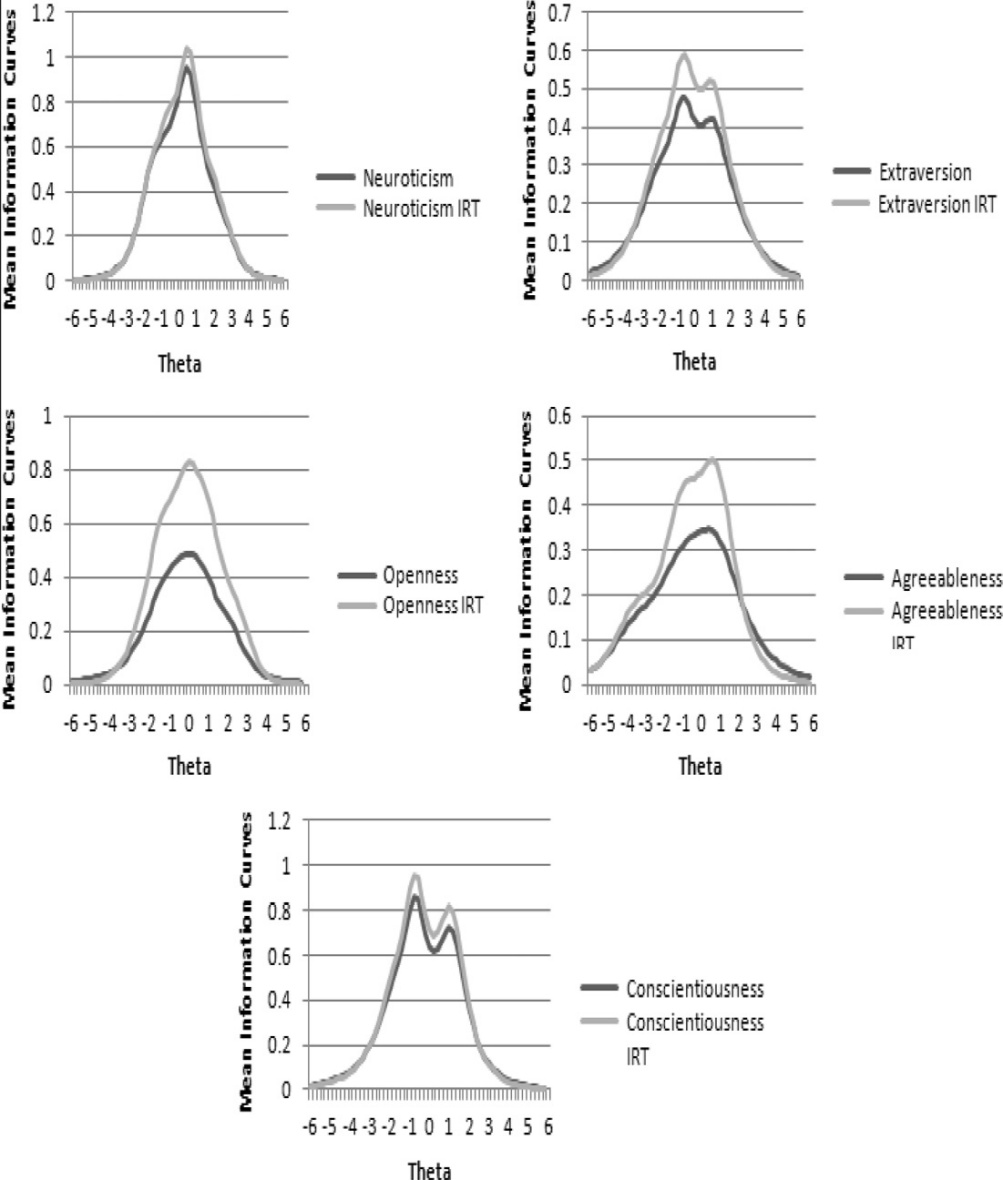
Mean information curves for each scale before and after IRT.

The information function analysis was run with the less discriminatory items removed. Information curves are sensitive to scale length, therefore following the method of Samuel, Simms, Clark, Livesley, and Widiger (2010) the IICs were averaged to control for different scale lengths. These ‘mean information curves’ demonstrated that the scales provided more information when the poorly performing items were removed but without changing where along the latent trait continuum most information was provided (see Fig. 2).

### 3.2 External validity

To ascertain whether the non-discriminatory items could be removed from the NEO-FFI without meaningfully reducing external validity, the factors were individually correlated or regressed onto the external measures. Correlations and regressions before and after IRT were compared. Results are reported for the general factors (see Table 3).

The associations demonstrated that for the majority of the scales removing items was not detrimental to external validity. As hypothesised more neurotic individuals had lower levels of well-being, whilst more extraverted and conscientious people had greater well-being. Additionally, more agreeable and extraverted participants rated their friendships as more satisfying. However, although Openness was somewhat related with academic achievement, Conscientiousness was not. Interestingly, it appeared that Neuroticism and Conscientiousness were significantly related with friendships, whilst Openness was positively associated with well-being, which had not been hypothesised.

In general, the differences between the correlations before and after IRT were small and for all of the five scales the differences were not significant (see Table 4). However the results of the Openness scale validation were mixed. Before IRT, Openness was significantly correlated with some aspects of school performance whereas it was not afterwards; nevertheless the difference in magnitude of the associations was small.

## 4 Discussion

The analysis demonstrated that many items (*n* = 19) failed to discriminate to an acceptable level in this adolescent population. The majority (*n* = 16) being from the Extraversion, Agreeableness and Openness scales. The removed items did not appear to greatly contribute to the measurement of personality; correlating the external criterion with the traits demonstrated that removing non-discriminatory items did not, for the majority of the scales, effect external validity. One caveat was the Openness scale, whose performance differed before and after IRT. Additionally, the external correlations illustrated that scoring low on Neuroticism and higher on the other four traits may help adolescents achieve greater levels of competence across different domains of functioning. Such a personality profile may be of value in studies of adolescent development and contribute to understanding individual differences in treatment response for common mental illnesses in the adolescent years.

IRT identified a large minority of items that did not discriminate well. Studies of the NEO-FFI in adolescents have found many items do not load sufficiently on any factor or cross load onto unintended factors. This is particularly the case for Extraversion, Agreeableness and Openness, whilst Neuroticism and Conscientiousness tend to perform better (Parker & Stumpf, 1998; Sneed et al., 2002). The results of the current study suggest these findings are likely due to a lack of discriminatory power of many items, suggesting they are not measuring the underlying latent traits strongly.

Previous studies report few difficulties with item comprehension (De Fruyt, Mervielde, Hoekstra, & Rolland, 2000; McCrae et al., 2005), therefore it is unlikely the lack of discrimination reflects a limited understanding of the questions. Perhaps many of the trait indicators fail to discriminate appropriately on the latent traits because the items are not referencing ideas or behaviours that are relevant to the cultural milieu of adolescents (Sneed et al., 2002).

Additionally, the threshold data demonstrated that for the majority of items only people over three standard deviations away from the population mean responded to the categories ‘*strongly agree*’ and/or ‘*strongly disagree*’. Compared to published norms this sample had lower Neuroticism and higher Agreeableness, which may somewhat explain these results. However it did not differ on Openness, Extraversion or Conscientiousness suggesting for the current trait indicators these response categories only have limited utility for most adolescents in the general population.

Thalmayer et al. (2011) found brief personality questionnaires had similar levels of predictive ability and argued that scales comprised of a few high-validity items may obtain equal predictive validity to those of their longer counterparts. The result from the present study support these assertions as the more discriminating items allowed a reduction in scale length that was just as externally valid.

Nonetheless, the Agreeableness and Openness items discriminated poorly; with IRT affecting the Openness scale’s performance. Thus use of these shortened scales must be done so with caution. As half of the indicators were not strongly measuring the latent traits questions arise as to what constructs these scales may be evaluating. Indeed, convergence between the NEO-FFI Agreeableness scale and social desirability measures have been reported in adults (Stöber, 2001) and there is evidence suggesting Openness measures a trait related to intellectual ability (Ferguson & Patterson, 1998), indicating there may be some confusion of measurement.

### 4.1 Limitations

A limitation of the present study is whether the sample is representative of British adolescents. The return rate of 43.8% means the majority of adolescents from the ROOTS cohort did not participate. A comparison to norms published by Costa and McCrae (1992) shows this sample to be more agreeable and less neurotic, suggesting they are more emotionally stable, altruistic and willing to help others. More research would help to elucidate whether these norms are appropriate for British adolescents or if this is a reflection of idiosyncratic properties of this sub-sample. Further replication would also clarify the generalisability of the IRT analysis and discern the reliability of the *a* and *b* parameters in UK adolescents.

The measures used for the external validation of the NEO-FFI were collected earlier than the personality information, rather than concurrently. The well-being scale measures within a 2 week period and personality is apt to some change over adolescence (McCrae et al., 2002), however the friendship scale considers a 12 month period and the GCSE results would not change. Nonetheless, this could feasibly influence the results of the external validity analysis. Even so, the personality traits correlated with the measures as hypothesised, therefore it is unlikely this time difference unduly affected the results.

## 5 Conclusions

Personality is consistently used as an important explanatory factor in a large number of studies. The present study provided an item-level analysis allowing for a thorough examination of the assumed personality factors, highlighting scale strengths but also weaknesses. This was particularly the case for the Openness scale, which performed poorly and was influenced to the greatest degree by item removal. The results suggest that for adolescents many items considered as measuring components of personality are not discriminating along the latent traits to a high degree. These cannot therefore be used as reliable indicators, hindering internal validity. The results suggests future directions for testing and refinement, especially with the Agreeableness and Openness scales, which may need more development and testing before they can be used reliably in adolescent populations. Overall, the present study suggests the use of briefer more efficient personality measures with highly discriminating items may be more internally valid and achieve equal external validity.

## Figures and Tables

**Table 1:** Variance and goodness of fit indices for unidimensionality assessment (a) before modifications and (b) after modifications.

	Neuroticism		Extraversion		Openness		Agreeableness		Conscientiousness	
	*a*	*b*	*a*	*b*	*a*	*b*	*a*	*b*	*a*	*b*
Variance explained	.49	.42	.43	.38	.20	.23	.48	.23	.29	.25
CFI	.942	.961	.895	.931	.914	.960	.854	.926	.920	.960
RMSEA	.109	.093	.100	.084	.094	.065	.101	.073	.124	.092

**Table 2:** Item parameters for the NEO-FFI items.

		*A*	*a*1	*a*2	*a*3	*a*4	*a*5	*b*1	*b*2	*b*3	*b*4
*Neuroticism*											
N1	Worrier (R)	1.41	1.19					−3.49	−1.18	0.00	2.66
N2	Feel inferior	1.56						−2.78	−0.13	1.58	4.39
N3	Go to pieces	1.69						−3.01	−0.49	0.44	3.00
N4	Lonely (R)	1.89						−3.37	−0.06	1.19	3.81
N5	Tense	2.73		0.52	1.52			−4.11	1.12	3.13	7.54
N6	Worthless	3.29		−0.35				−2.25	1.15	2.36	5.96
N7	Fearful (R)	2.27	0.98		0.81			−4.50	−0.42	1.17	4.57
N8	Angry	1.17						−3.15	−0.10	1.07	3.39
N9	Discouraged	2.03				0.81		−3.54	−0.18	1.45	4.49
**N10**	**Sad (R)**	**0.71**						**−2.79**	**−0.38**	**0.43**	**2.04**
N11	Helpless	2.87				2.23		−4.11	1.06	3.52	7.76
N12	Ashamed	1.65						−1.63	0.78	1.79	4.26

*Extraversion*											
E1	People around	1.19	0.82					−5.77	−2.82	−0.13	2.50
**E2**	**Laugh easily**	**1.08**						**−6.84**	**−4.13**	**−2.07**	**1.05**
**E3**	**‘Light hearted’ (R)**	**0.84**		**0.82**				**−4.01**	**−1.86**	**−0.02**	**2.60**
E4	Enjoy talking	1.58							−5.11	−2.46	0.96
E5	Like action	1.85	1.56		1.83				−4.65	−1.14	3.91
**E6**	**Do things alone (R)**	**1.10**						**−3.65**	**−1.55**	**0.36**	**3.46**
E7	Bursting with energy	1.31						−4.55	−1.35	0.75	3.38
E8	Cheerful	2.81				1.34		−8.24	−6.17	−2.14	2.81
E9	Optimist (R)	1.71		0.77		0.86		−5.70	−3.01	−1.06	2.37
**E10**	**Fast-paced**	**0.96**			**0.77**			**−5.31**	**−1.61**	**0.36**	**2.97**
E11	Active	1.17						−4.03	−1.99	−0.31	2.04
**E12**	**Leader of others (R)**	**0.85**						**−3.50**	**−1.01**	**0.69**	**2.79**

*Openness*											
**O1**	**Daydreaming (R)**	**0.83**						**−2.92**	**−0.96**	**0.34**	**2.53**
**O2**	**Find the right way (R)**	**0.01**							**−1.49**	**1.48**	**3.28**
O3	Patterns	2.92	1.71					−4.29	−1.55	1.04	4.47
**O4**	**Controversial speakers (R)**	**0.99**						**−4.62**	**−3.12**	**−1.05**	**1.03**
O5	Poetry (R)	2.20		2.09				−3.31	−0.54	1.58	4.83
**O6**	**Foreign foods**	**0.60**						**−3.00**	**−1.47**	**−0.49**	**1.40**
**O7**	**Notice moods (R)**	**0.68**						**−4.39**	**−1.55**	**−0.14**	**2.34**
**O8**	**Religious authorities (R)**	**0.13**						**−3.57**	**−2.44**	**−0.94**	**0.31**
O9	Wave of excitement	2.41	1.49	1.90				−3.29	−0.12	1.95	5.86
O10	Interest in speculating (R)	1.67						−4.33	−2.29	−0.63	1.88
O11	Curiosity	1.47							−3.91	−1.75	1.57
O12	Enjoy theories	1.91						−4.76	−1.49	0.19	2.75

*Agreeableness*											
A1	Respectful	1.58	3.00					−11.22	−9.26	−6.01	0.25
A2	Arguments (R)	1.27						**−5.80**	**−2.97**	**−1.64**	**1.21**
A3	Egotistical (R)	1.70		0.84				−6.18	−2.81	−1.48	2.32
**A4**	**Co-operate**	**0.68**						**−4.98**	**−2.23**	**−0.79**	**1.73**
A5	Cynical (R)	1.20						−3.61	−1.11	0.57	2.95
**A6**	**Take advantage (R)**	**0.81**						**−2.75**	**−0.49**	**0.49**	**3.24**
**A7**	**People like me**	**0.87**							**−3.98**	**−1.83**	**1.83**
A8	Calculating (R)	1.62		0.67				−6.88	−3.50	−2.13	1.22
**A9**	**Hot-headed (R)**	**0.90**						**−3.54**	**−1.25**	**0.43**	**3.10**
A10	Thoughtful	1.39	1.61						−6.59	−4.73	0.91
**A11**	**Don’t like people (R)**	**0.95**						**−3.19**	**−1.13**	**0.07**	**2.44**
**A12**	**Manipulate people (R)**	**1.16**						**−4.24**	**−1.68**	**−0.45**	**1.76**

*Conscientiousness*											
**C1**	**Neat**	**1.06**	**1.16**					**−3.22**	**−0.61**	**1.18**	**3.60**
C2	Pace myself	2.08						−5.14	−1.67	−0.25	3.26
**C3**	**Methodical (R)**	**0.95**						**−5.18**	**−1.96**	**−0.21**	**2.65**
C4	Perform thoroughly	1.91							−4.04	−1.88	1.68
C5	Clear goals	1.82						−4.86	−1.55	0.13	3.34
C6	Waste time (R)	1.24						−2.31	0.23	1.32	4.07
C7	Accomplish goals	3.03			1.34	0.42		−10.79	−6.12	−2.95	2.31
C8	Counted on	2.07		1.62			1.88	−10.10	−6.00	−2.72	3.13
C9	Reliable (R)	1.44					1.71	−6.79	−1.28	−0.27	3.88
C10	Productive	3.55		1.67		0.62		−11.70	−7.10	−2.38	4.35
C11	Organised (R)	1.96	1.11					−4.09	−1.15	0.29	4.11
C12	Strive for excellence	2.09			1.38			−9.04	−4.02	−1.33	1.61


*Note: A* = general factor, *a* = group factor discrimination parameters; representing the slope of the curve at the inflection point, *b* = threshold parameters for the general factor; the point where the response curves for each response category intersect. Items in bold fail to achieve at least moderate to high discrimination (*a* < 1.17).

**Table 3:** NEO-FFI correlations and regressions with the external measures (a) before IRT and (b) after IRT.

	Neuroticism		Extraversion		Openness		Agreeableness		Conscientiousness	
	a	b	a	b	a	b	a	b	a	b
Well-being	−.24⁎⁎	−.24⁎⁎	.26⁎⁎	.23⁎⁎	.11⁎	.10⁎	.06	.05	.14⁎⁎	.15⁎⁎
Friendship	−.20⁎⁎	−.19⁎⁎	.27⁎⁎	.25⁎⁎	.05	.04	.15⁎⁎	.14⁎	.18⁎⁎	.18⁎⁎
School										
Entries	−.10⁎	−.10⁎	.09	.06	.12⁎	.09	.05	.02	.01	.01
A^∗^–C	.11⁎	−.11⁎	.06	.03	.12⁎	.09	.05	.02	.00	.00
D–G	.08	.09	−.02	.00	−.09	−.07	−.04	−.01	.00	.01

⁎ *p* < .05.

⁎⁎ *p* < .01.

**Table 4:** Steiger’s *z*-test comparing the NEO-FFI correlations before and after IRT.

	Well-being	Friendship	School		
			Entries	A–C	D–G
Neuroticism	.00	.16	.00	.00	.15
Extraversion	.48	.32	.46	.46	.31
Openness	.15	.15	.46	.46	.31
Agreeableness	.15	.16	.46	.46	.46
Conscientiousness	.16	.00	.00	.00	.15

*Note:* Values >1.96 are significant, *p* = .05.

## References

[b0005] Baker F.B. (2001). The basics of item response theory.

[b0010] Chamorro-Premuzic T., Furnham A. (2003). Personality predicts academic performance: Evidence from two longitudinal university samples. Journal of Research in Personality.

[b0015] Costa P.T., McCrae R.R. (1992). Revised NEO personality inventory (NEO-PI-R) and NEO five factor inventory (NEO-FFI) professional manual.

[b0020] Costello E.J., Copeland W., Angold A. (2011). Trends in psychopathology across the adolescent years: What changes when children become adolescents, and when adolescents become adults?. Journal of Child Psychology and Psychiatry.

[b0025] De Fruyt F., De Bolle M., McCrae R.R., Terracciano A., Costa P.T. (2009). Assessing the universal structure of personality in early adolescence: The NEO-PI-R and NEO-PI-3 in 24 cultures. Assessment.

[b0035] De Fruyt F., Mervielde I., Hoekstra H.A., Rolland J.P. (2000). Assessing adolescents’ personality with the NEO PI-R. Assessment.

[b0030] De Fruyt F., van Leeuwen K., de Bolle M., de Clercq B. (2008). Sex differences in school performance as a function of Conscientiousness, imagination and the mediating role of problem behaviour. European Journal of Personality.

[b0165] Embretson S.E., Reise S.P. (2000). Item response theory for psychologists.

[b0045] Ferguson E., Patterson F. (1998). The five factor model of personality: Openness as a distinct but related construct. Personality and Individual Differences.

[b0150] Goodyer I.M., Croudace T., Dunn V., Herbert J., Jones P.B. (2010). Cohort profile: Risk patterns and processes for psychopathology emerging during adolescence: The ROOTS project. International Journal of Epidemiology.

[b0055] Goodyer I.M., Wright C., Altham P.M. (1989). Recent friendships in anxious and depressed school age children. Psychological Medicine.

[b0060] Hřebíčková M., Urbánek T., Čermák I., Szarota P., Ficková E., Orlická L., McCrae R.R., Allik J. (2002). The NEO five-factor inventory in czech, polish, and slovak contexts. The five factor model of personality across cultures.

[b0065] Masten A.S., Coatsworth J.D., Neemann J., Gest S.D., Tellegen A., Garmezy N. (1995). The structure and coherence of competence from childhood through adolescence. Child Development.

[b0070] McCrae R.R., Costa P.T., Martin T.A. (2005). The NEO-PI-3: A more readable revised NEO personality inventory. Journal of Personality Assessment.

[b0075] McCrae R.R., Costa P.T., Terracciano A., Parker W.D., Mills C.J., De Fruyt F. (2002). Personality trait development from age 12 to age 18: Longitudinal, cross-sectional, and cross-cultural analyses. Journal of Personality and Social Psychology.

[b0155] Muthén L.K., Muthén B.O. (1998). M*plus* user’s guide.

[b0085] Parker W.D., Stumpf H. (1998). A validation of the five-factor model of personality in academically talented youth across observers and instruments. Personality and Individual Differences.

[b0090] Reise S.P., Henson J.M. (2000). Computerization and adaptive administration of the NEO-PI-R. Assessment.

[b0095] Reise S.P., Morizot J., Hays R.D. (2007). The role of the bifactor model in resolving dimensionality issues in health outcomes measures. Qualitative Life Research.

[b0160] Samejima F. (1969). Estimation of latent ability using a response pattern of graded scores. Psychometrika Monograph.

[b0105] Samuel D.B., Simms L.J., Clark L.A., Livesley W.J., Widiger T.A. (2010). An item response theory integration of normal and abnormal personality scales. Personality Disorders: Theory, Research, and Treatment.

[b0110] Scholte R.H.J., van Aken M.A.G., van Lieshout C.F.M. (1997). Adolescent personality factors in self-ratings and peer nominations and their prediction of peer acceptance and peer rejection. Journal of Personality Assessment.

[b0115] Selfhout M., Burk W., Branje S., Denissen J., van Aken M., Meeus W. (2010). Emerging late adolescent friendship networks and big five personality traits: A social network approach. Journal of Personality.

[b0120] Siegler I.C., Brummett B.H. (2000). Associations among NEO personality assessments and well-being at midlife: Facet-level analyses. Psychology and Aging.

[b0125] Sneed C.D., Gullone E., Moore S. (2002). Reliability and factor structure of the NEO-five-factor inventory for Australian adolescents. Behaviour Change.

[b0130] Soto C.J., John O.P., Gosling S.D., Potter J. (2008). The developmental psychometrics of big five self-reports: Acquiescence, factor structure, coherence, and differentiation from ages 10 to 20. Journal of Personality and Social Psychology.

[b0135] Stöber J. (2001). The social desirability scale-17 (SDS-17) convergent validity, discriminant validity, and relationship with age. European Journal of Psychological Assessment.

[b0140] Tennant R., Fishwick R., Platt S., Joseph S., Stewart-Brown S. (2006). Monitoring positive mental health in Scotland: Validating the Affectometer 2 scale and developing the Warwick-Edinburgh Mental Well-being Scale for the UK.

[b0145] Thalmayer A.G., Saucier G., Eigenhuis A. (2011). Comparative validity of brief to medium-length big five and big six personality questionnaires. Psychological Assessment.

